# Age-related changes in neural responses to sensory stimulation in autism: a cross-sectional study

**DOI:** 10.1186/s13229-023-00571-4

**Published:** 2023-10-11

**Authors:** Melis E. Cakar, Kaitlin K. Cummings, Susan Y. Bookheimer, Mirella Dapretto, Shulamite A. Green

**Affiliations:** 1 Neuroscience Interdepartmental Program, Ahmanson Lovelace Brain Mapping Center, University of California Los Angeles, 660 Charles E. Young Drive South, Los Angeles, CA 90095 USA; 2 Department of Psychology and Neuroscience, The University of North Carolina at Chapel Hill, Chapel Hill, USA; 3 Department of Psychiatry and Biobehavioral Sciences, University of California Los Angeles, Los Angeles, USA; 4 Jane and Terry Semel Institute for Neuroscience and Human Behavior, University of California Los Angeles, Los Angeles, USA

**Keywords:** Sensory over-responsivity, Sensory processing, Autism spectrum disorder, Development, fMRI, Neural activity

## Abstract

**Background:**

Sensory over-responsivity (SOR) is an impairing sensory processing challenge in autism spectrum disorder (ASD) which shows heterogenous developmental trajectories and appears to improve into adulthood in some but not all autistic individuals. However, the neural mechanisms underlying interindividual differences in these trajectories are currently unknown.

**Methods:**

Here, we used functional magnetic resonance imaging (fMRI) to investigate the association between age and neural activity linearly and nonlinearly in response to mildly aversive sensory stimulation as well as how SOR severity moderates this association. Participants included 52 ASD (14F) and 41 (13F) typically developing (TD) youth, aged 8.6–18.0 years.

**Results:**

We found that in pre-teens, ASD children showed widespread activation differences in sensorimotor, frontal and cerebellar regions compared to TD children, while there were fewer differences between ASD and TD teens. In TD youth, older age was associated with less activation in the prefrontal cortex. In contrast, in ASD youth, older age was associated with more engagement of sensory integration and emotion regulation regions. In particular, orbitofrontal and medial prefrontal cortices showed a nonlinear relationship with age in ASD, with an especially steep increase in sensory-evoked neural activity during the mid-to-late teen years. There was also an interaction between age and SOR severity in ASD youth such that these age-related trends were more apparent in youth with higher SOR.

**Limitations:**

The cross-sectional design limits causal interpretations of the data. Future longitudinal studies will be instrumental in determining how prefrontal engagement and SOR co-develop across adolescence.

**Conclusions:**

Our results suggest that enhanced recruitment of prefrontal regions may underlie age-related decreases in SOR for a subgroup of ASD youth.

**Supplementary Information:**

The online version contains supplementary material available at 10.1186/s13229-023-00571-4.

## Background

Autism spectrum disorder (ASD) is a neurodevelopmental disorder marked by social, communicative, and behavioral features that emerge in early childhood [1, 2]. Sensory features are part of the core diagnostic criteria for ASD and are highly prevalent, with an estimated rate of over 90% among ASD youth [3, 4]. More than half of ASD youth experience sensory over-responsivity (SOR), a particularly impairing sensory issue characterized by an extreme negative response to or avoidance of sensory stimulation [5, 6]. SOR often emerges in early childhood [5, 7, 8] and is associated with elevated anxiety as well as internalizing and externalizing problems [9, 10]. While SOR may improve with age [11, 12], little is known about the neural basis of developmental changes in SOR symptoms.

The ASD population is highly heterogenous in genetics, brain anatomy and function, behavioral abilities, challenges and strengths, and developmental trajectories [13–16]. Autistic individuals show interindividual heterogeneity in developmental changes also in sensory symptoms broadly [17, 18], but different types of sensory processing challenges (e.g., SOR, sensory *under*-responsivity, and sensory seeking) display unique developmental patterns both within [19] and outside of autism [20]. Focusing on developmental changes in *SOR* specifically, Green et al. [7] found that parent-reported SOR severity remained stable in ASD toddlers (aged 1.5–2.8 years) in a one-year longitudinal study. Baranek et al., [19] corroborated these findings in older children, similarly reporting no significant decline in parent-reported SOR with age in ASD in a three-year longitudinal study of children aged 2–12 years. While these findings suggest that SOR features may remain stable across childhood, there are few studies that continue to track SOR into adolescence and adulthood. A few cross-sectional studies suggest that SOR may improve into adulthood in some, but not all individuals with ASD: Two cross-sectional studies using caregiver-report [11, 12] suggest that SOR severity and prevalence may be reduced in adolescence and adulthood compared to in middle childhood. Using a different approach, Tavassoli et al. [21] assessed SOR via self-report and found that autistic adults reported more SOR compared to neurotypical peers, indicating that autistic individuals overall show elevated SOR compared to neurotypicals into adulthood. Taken together, these results indicate that developmental trajectories of SOR may be heterogenous within the ASD population. As suggested by cross-sectional studies, some ASD youth may show improvements in SOR features with age, but SOR likely continues to be a challenge into adulthood in comparison with neurotypical individuals. However, the neural basis of age-related trajectories in SOR and how these trajectories compare to typically developing (TD) youth remain unclear.

A few studies have shown both structural and functional brain differences associated with severity of sensory challenges, including Ecker et al. [22] and Green et al. [23]. In particular, functional magnetic resonance imaging (fMRI) studies have shown that ASD youth display heightened activation in response to sensory stimulation in sensory cortices and subcortical regions, including the amygdala and thalamus [24, 25]. Importantly, higher activation in sensory-limbic regions was associated with more severe SOR in ASD. Compared to ASD youth with SOR, ASD youth without SOR showed stronger negative functional connectivity between the amygdala and orbitofrontal cortex (OFC) during sensory stimulation, suggesting that the prefrontal cortex (PFC) may be involved in a compensatory mechanism for sensory regulation in autistic youth without SOR [23, 25]. However, despite the behavioral evidence suggesting changes in SOR across adolescence and into adulthood, prior neuroimaging research averaged neural responses across a large age range, which may lead to the assumption that ASD youth process sensory signals similarly across ages. This prior research hence did not allow identification of age-specific mechanisms of sensory reactivity or regulation. For example, PFC-amygdala circuitry is involved in emotion regulation and matures structurally and functionally throughout adolescence [26–29]. Importantly, the inverse coupling between PFC and amygdala forms in early adolescence and strengthens into adulthood, and is associated with a decrease in amygdala reactivity as well as lower anxiety [27, 29]. These studies indicate that, in typical development, the regulatory function of the PFC on the amygdala may come online at the onset of adolescence. While sensory regulation through the PFC could potentially be a mechanism for age-related attenuation of SOR seen in some ASD youth, the association between age and neural responses to sensory signals has not yet been thoroughly examined. Uncovering these mechanisms is key to developing targeted treatments for ASD youth whose SOR symptoms persist into school-age and later developmental stages.

In the current study, we investigated the effect of age on the neural mechanisms of sensory responsivity. First, we aimed to assess neural differences during sensory processing cross-sectionally in younger versus older youth to examine whether previously seen neural differences between ASD and TD youth were consistent across age groups. Second, we investigated age as a continuous predictor linearly and nonlinearly to characterize neural mechanisms that may account for age-related changes in sensory responsivity in ASD and in TD youth. Third, given the evidence showing neural heterogeneity among ASD youth with high vs. low SOR severity [23, 25], we examined how SOR severity might affect the relationship between age and neural responsivity.

## Methods

### Participants

Participants consisted of 52 ASD (14F) and 41 TD (13F) participants, with an age range of 8.6 *–* 18.0 years (Table 1). Written informed consent was obtained from all parents and from children who were 13 years or older; written assent was given by participants who were younger than 13 years. ASD diagnosis was confirmed with the Autism Diagnostic Interview-revised (ADI-R; [25]), Autism Diagnostic Observation Schedule—second edition (ADOS-2; [26]) and clinical judgment. Full-scale IQ (FSIQ) ranged between 78 and 146 on the Wechsler Abbreviated Scales of Intelligence (WASI; [27]). FSIQ was significantly lower in the ASD group (see Table 1) and was thus included as a covariate in subsequent categorical age (i.e., pre-teens and teens), age-by-diagnosis and age^2^-by-diagnosis analyses (see Additional file 1: for analyses without IQ as a covariate). Diagnostic groups did not differ significantly in age, sex, race, or ethnicity (Table 1). Further information on medication use and co-occurring conditions in the ASD group can be found in Additional file 1; Table S1 and S2. All study procedures were approved by the University of California, Los Angeles, Institutional Review Board. A subset of these participants (ASD: *n* = 42; TD: *n* = 27) were included in a prior study that examined sensory habituation in a few specific brain regions, and the effect of age was not addressed [23]

**Table 1:** Descriptive statistics

	ASD (mean ± SD)	TD (mean ± SD)	*t or χ*^*2*^
*N*	52	41	–
Pre-teens (< 13 years of age)	26	22	–
Teens (> 13 years of age)	26	19	–
Age (years)	13.44 ± 2.79	13.01 ± 3.01	*p* = 0.48
Sex (females)	14	13	*p* = 0.61
*Ethnicity*			
Hispanic or Latino/a	17	13	*p* = 0.92
Not Hispanic or Latino/a	35	28	
*Race*			
Asian	3	7	*p* = 0.47^†^
Black or African American	3	3	
White	29	20	
More than One Race	15	9	
Unknown or Not Reported	2	2	
*Scanner motion*			
Mean absolute motion (mm)	0.44 ± 0.23	0.43 ± 0.22	*p* = 0.88
Mean relative motion (mm)	0.16 ± 0.13	0.15 ± 0.13	*p* = 0.61
Number of outlier volumes	27.63 ± 17.27	19.83 ± 14.56	*p* = 0.02*
WASI full-scale IQ	105.37 ± 15.87	113.56 ± 13.20	*p* = 0.009*
SOR total score	9.12 ± 7.34	1.65 ± 3.84	*p* < 0.001*^‡^

^†^ Fisher’s exact test was used to assess independence of the variables. ^‡^ One TD participant was excluded due to missing SOR score. *SD* Standard deviation, *ASD* Autism spectrum disorder, *TD* Typically developing, *WASI* Wechsler Abbreviated Scale Intelligence, *SOR* Sensory over-responsivity. Higher scores of SOR indicate more severe SOR

**Table 2:** Cluster peak coordinates

Contrast	Region	Additional regions covered	Voxels	Z-max	x	y	z
Categorical age analysis							
ASD pre-teens	(Right) Auditory temporal cortex	(Right) Insular cortex, temporal pole, supramarginal gyrus (anterior and posterior), sensorimotor cortex, superior parietal lobule, frontal operculum, posterior cingulate gyrus, supplementary motor cortex, frontal cortex, putamen	11,174	7.13	50	− 28	16
	(Left) Auditory temporal cortex	(Left) Insular cortex, temporal pole, posterior supramarginal gyrus (anterior and posterior), sensorimotor cortex, hippocampus	5856	8.7	− 32	− 34	14
	(Right) Cerebellar lobule VIIIA	Cerebellar (bilateral) lobules VI, VIIB and Crus I and II; and (left) lobules V, VIIIA and B, and X	5687	7.06	30	− 60	− 58
	(Left) Dorsolateral prefrontal cortex/frontal pole/ white matter	–	886	5.6	-30	34	18
	Brainstem	(Right) Amygdala, hippocampus, thalamus	549	4.89	6	− 40	− 10
TD pre-teens	(Right) Auditory temporal cortex	(Right) Insular cortex, temporal pole, supramarginal gyrus (anterior and posterior), sensorimotor cortex, right amygdala	4377	6.18	40	-26	20
	(Left) Auditory temporal cortex	(Left) Insular cortex, temporal pole, sensorimotor cortex, supramarginal gyrus (anterior)	3470	5.76	− 50	− 6	8
	(Right) Superior parietal lobule	(Right) Precentral gyrus, postcentral gyrus,	1016	5.14	26	− 40	66
	(Left) Cerebellar lobule VIIIB	(Left) Cerebellar lobules VIIB, VIIIA and Crus II	863	6.17	− 26	− 44	− 52
	(Right) Ventrolateral prefrontal cortex/frontal pole	-	399	5.3	34	38	6
ASD > TD pre-teens *(masked by activation in ASD pre-teens)*	(Right) Cerebellar lobule VI	(Right) Cerebellar lobules VIIIA and VIIB	221	3.69	34	− 40	− 38
	(Right) Dorsolateral prefrontal cortex/frontal pole	–	189	3.57	18	34	34
	(Right) Superior parietal lobule/postcentral gyrus	–	175	3.5	26	− 44	64
	(Right) Cerebellar Crus I	Right cerebellar lobule VI	100	3.92	38	− 50	− 32
	(Left) Dorsolateral prefrontal cortex	–	98	3.48	− 40	30	24
	Posterior cingulate gyrus	–	74	4.02	− 10	− 32	40
	(Left) Superior parietal lobule	–	25	2.9	− 18	-52	68
	Frontal pole/ ventromedial prefrontal cortex	–	16	3.34	-14	48	− 16
ASD > TD pre-teens *(masked by deactivation in TD pre-teens)*	Precuneus/superior lateral occipital cortex/sensorimotor cortex (left postcentral and right precentral gyrus)	Superior parietal lobule	1108	3.8	20	− 60	60
	(Left) Precentral/postcentral gyrus	–	452	4.02	− 40	− 22	54
	(Right) Dorsolateral prefrontal cortex/frontal pole	–	425	4.09	32	28	30
	(Left) Dorsolateral prefrontal cortex	–	395	3.77	-40	18	40
	(Left) Superior lateral occipital cortex/angular gyrus	–	373	3.48	− 42	− 82	30
	(Right) Cerebellar lobule VI	(Right) Crus I, Lobules I-V	357	4.03	40	− 48	− 30
	Ventromedial prefrontal cortex	–	330	3.86	− 12	56	− 14
	Posterior cingulate gyrus	–	179	4.02	− 10	− 32	40
	Anterior cingulate gyrus	–	18	2.85	6	32	16
	(Right) Cerebellar lobule VIIIA	–	14	3.07	30	-44	-46
	(Right) Cerebellar lobule VIIB and Right Crus II	–	10	2.82	44	− 42	− 46
ASD teens	(Right) Auditory temporal cortex	(Right) Insular cortex, temporal pole, supramarginal gyrus (anterior), sensorimotor cortex, orbitofrontal cortex, right putamen	4981	6.53	46	− 22	16
	(Left) Insular cortex	(Left) Auditory temporal cortex, temporal pole, supramarginal gyrus (anterior and posterior), sensorimotor cortex, left amygdala	4549	6.34	− 42	− 8	− 2
	(Right) Cerebellar lobule VIIB	Bilateral cerebellar VIIIA and Crus II; left cerebellar lobules V, VI, VIIB, VIIIB, and X	1818	6.07	12	− 76	− 50
	(Right) Precentral gyrus	(Right) superior frontal gyrus, postcentral gyrus, superior parietal lobule and posterior cingulate gyrus	1558	5.7	18	-16	58
	Brainstem	(Left) Posterior parahippocampal gyrus	535	5.6	-6	-36	-12
TD teens	(Right) Insular cortex	(Right) Auditory temporal cortex, temporal pole, postcentral gyrus, supramarginal gyrus (anterior), orbitofrontal cortex, frontal pole, putamen, pallidum, thalamus, and amygdala	5316	6.33	36	-16	16
	(Left) Auditory temporal cortex	(Left) Insular cortex, temporal pole, postcentral gyrus, supramarginal gyrus (anterior), orbitofrontal cortex, putamen, and amygdala	3204	5.01	-56	-24	14
	(left) Cerebellar lobule VIIIA	(Left) Cerebellar lobules VIIIB, VIIB, Crus I and Crus II	534	3.97	-28	-60	-48
	(Right) Postcentral gyrus	(Right) Precentral gyrus, superior parietal lobule	529	4.51	34	-36	66
ASD > TD teens *(masked by activation in ASD teens)*	Hippocampus/white matter		38	3.79	30	-38	4
	Posterior cingulate/lingual gyrus	-	12	3.45	10	-46	2
ASD > TD teens *(masked by deactivation in TD teens)*	(Right) Posterior cingulate gyrus	(Right) Lingual gyrus, cerebellar lobule V, hippocampus/thalamus	469	3.94	14	-42	-2
Continuous age analysis							
ASD positive	(Left) Insula/ temporal pole/ orbitofrontal cortex	-	458	4.34	-40	12	-16
ASD negative	Precuneus	Superior lateral occipital cortex	549	3.62	12	-54	54
TD negative	(Right) Frontal pole/ventrolateral prefrontal cortex	-	486	3.25	40	44	6
Continuous Age^2^ Analysis							
ASD positive	Medial prefrontal cortex/ superior frontal gyrus	Left middle frontal gyrus/dorsolateral prefrontal cortex, supplementary motor cortex	1159	3.83	− 4	44	42
	(Left) Orbitofrontal cortex	(Left) Temporal pole, inferior frontal gyrus pars triangularis, frontal operculum cortex	464	4.02	− 38	26	− 14
	Occipital pole	Intracalcarine cortex	459	3.49	− 8	− 104	0
	(Right) Dorsolateral prefrontal cortex/middle frontal gyrus	–	398	3.52	42	22	52
TD negative	Medial prefrontal cortex / paracingulate gyrus/superior frontal gyrus	–	662	3.8	6	52	20
ASD > TD *(masked by ASD Age*^*2*^* positive)*	Medial prefrontal cortex / paracingulate gyrus/superior frontal gyrus	Supplementary motor cortex	1622	4.57	6	52	20
	(Left) Orbitofrontal cortex	(Left) Temporal pole, inferior frontal gyrus part triangularis and part orbital, frontal operculum cortex	757	4.49	− 36	24	− 14
	(Left) Angular gyrus/white matter	(Left) Posterior supramarginal gyrus, temporooccipital middle temporal gyrus, lateral occipital cortex	465	4.59	− 42	− 52	12
ASD > TD *(masked by TD Age*^*2*^* negative)*	Medial prefrontal cortex/ paracingulate gyrus/ superior frontal gyrus		1181	4.57	6	52	20
	(Left) Orbitofrontal cortex	(Left) Temporal pole, inferior frontal gyrus pars triangularis, frontal operculum cortex	514	4.49	− 36	24	− 14
	(Left) Temporal pole	–	59	3.09	− 38	14	− 26
Age*SOR Interaction Analysis							
Positive	(Left) Temporal pole	(Left) Orbitofrontal cortex, ventromedial prefrontal cortex, and anterior parahippocampal gyrus	525	4.18	− 34	20	− 30
	(Left) Middle temporal gyrus	(Left) Inferior temporal gyrus	386	3.93	− 68	− 18	− 20
Negative	(Left) Precentral gyrus	–	446	4.11	− 20	− 18	72
Age^2^*SOR Interaction Analysis							
Positive	Superior frontal gyrus	Medial prefrontal cortex and right dorsolateral prefrontal cortex/ middle frontal gyrus	2094	4.06	14	22	66
	(Right) Caudate	Ventromedial prefrontal cortex, right putamen	1201	4.05	16	18	2
	(Left) Dorsolateral prefrontal cortex		926	4.45	− 32	40	44
	(Right) Angular gyrus/posterior supramarginal gyrus	Superior lateral occipital cortex	799	4.04	54	-54	38

The *Region* column indicates cluster peaks. Additional regions covered by the cluster beyond the peak are described under *Additional regions covered*

*Note.* X, y, and z refer to the left–right, anterior–posterior, and inferior–superior dimensions, respectively; Z refers to the Z-score at those coordinates. Voxels indicate cluster size. ASD > TD contrasts were masked by the ASD positive and TD negative within-group contrasts at z > 1.7, and masked clusters were reported separately (e.g., masked by activation in ASD teens or deactivation in TD teens). *ASD* Autism spectrum disorder, *TD* Typically developing youth, *SOR* Sensory over-responsivity

### Sensory over-responsivity measurement

As in previous studies with this population [25, 33–35], SOR severity was measured using the Sensory Processing 3-Dimension Scale (SP3D) Inventory [36], a rating scale whereby caregivers check off which of a number of sensory experiences bother their child. A total SOR score was calculated by summing the number of auditory, tactile, and visual items that parents indicated were bothersome to their child, with higher SOR scores indicating more severe SOR.

### MRI data acquisition

fMRI data were collected on a Siemens Prisma 3 Tesla MRI scanner. For each functional run, 706 multiband echo planar imaging volumes were acquired (TR = 720 ms, TE = 37 ms, flip angle = 52°, 208 mm FOV, 72 slices, voxel size = 2 × 2 × 2 mm). Participants were given magnet-compatible noise-cancelling headphones (i.e., Optoacoustics OptoActive II ANC) as well as earplugs to reduce scanner noise. Auditory stimuli were presented through noise-cancelling headphones.

### Sensory exposure paradigm

Participants experienced six blocks of auditory, tactile, and joint (i.e., simultaneous auditory and tactile) stimulation, each lasting 15 s. Total scan time was 8.5 min. The focus of the current study was on the joint condition as this condition more closely mimics multisensory exposure in real life and has been shown to best differentiate ASD and TD groups in prior studies (e.g., Green et al., [25]). Auditory stimulation involved pulsing pink and violet noise sounds. Tactile stimuli consisted of two different scratchy sponges which were matched for aversiveness level based on pilot testing and counterbalanced across participants. An experimenter rubbed participants’ inner left arm with a sponge attached to a rod at a rate of one stroke per second. The experimenters were trained to ensure reliability on the pressure of tactile stimulation (i.e., light pressure, just enough to avoid a tickle sensation), and to follow a timer. Participants were asked to focus on a central fixation cross throughout the task. The task consisted of 12.5 s of rest between trials as well as a 12.5-s initial and final rest. This fMRI paradigm was modeled on previous research by our group where the same [23] or a similar [24, 25, 37] sensory exposure paradigm was used to assess neural activation and functional connectivity during sensory exposure in ASD and TD youth.

### Analyses

#### fMRI data analyses

fMRI analyses were performed using the FMRIB Software Library (FSL; [33]), version 5.0.11. Statistical analyses were performed using the fMRI Expert Analysis Tool (FEAT; version 6.0) within FSL. Preprocessing steps included motion realignment to the mean image, spatial smoothing (Gaussian Kernel FWHM = 5 mm), high-pass temporal filtering (*t* > 0.01 Hz), and registration to the in-plane T2 image (6 degrees of freedom) to the MNI152 T1 2 mm brain (12 degrees of freedom). The motion criterion for exclusion was determined as greater than 1 mm mean absolute motion.

Fixed-effects models were run separately for single-subject level analyses and were subsequently integrated in a higher-level mixed-effects model to assess within- and between-group differences. Twelve motion parameters were included in single-subject level analyses as regressors. For further motion correction, outlier volumes were identified and regressed out for each participant using the fsl_motion_outliers tool. The ASD group had a significantly higher number of outlier volumes (Table 1). Mean absolute motion was significantly correlated with the number of outlier volumes (*r* = 0.58; *p* < 0.001) as well as with age (*r* = − 0.35, *p* < 0.001). Mean absolute motion was thus included as a covariate of no interest to control for motion in all of the subsequent analyses. In the current analyses, the joint condition was modeled with respect to fixation during rest. Group-level analyses were run with FSL’s Local Analysis of Mixed Effects State (FLAME 1 + 2; [34–36]). Results were corrected for multiple comparisons using Gaussian random-field theory (i.e., a type of family-wise error (FWE) rate correction) in FSL with a voxel-wise threshold of *z* > 2.3 and a cluster-corrected threshold of *p* < 0.05. Between-group contrasts were masked (post hoc) by within-group contrasts at a liberal threshold (z > 1.7; *p* < 0.05) to clarify which group was driving the differences (i.e., ASD > TD differences could be due to heightened activation in ASD, or heightened deactivation in TD; see below). All the analyses were also repeated with a more stringent voxel-wise threshold of z > 2.7 (see Additional file 1).

#### Neural response to sensory stimulation in older and younger ASD and TD youth

We aimed to interrogate diagnostic group (ASD versus TD) differences in sensory-evoked responses separately, in a group of younger and a group of older youth: pre-teenage children (“pre-teens”; aged 8.6–12.9 years) and teenagers (“teens”; aged 13.6–18.0 years). IQ was covaried in within-group as well as between-group analyses.

#### Age correlations with sensory-evoked neural activation

Next, to examine how age continuously related to neural responses to sensory stimulation in ASD and TD youth across the brain, age was entered as a bottom-up regressor and within-group correlations with age as well as an age-by-diagnosis effect were examined (Fig. 3). To further visualize the results, parameter estimates indexing neural activation were extracted from each cluster that had a significant relationship with age in the FEAT results (Fig. 3A) and were plotted against age in R (Fig. 3B).

To explore nonlinear changes with age, a separate analysis was conducted where age^2^ was entered as a bottom-up regressor in addition to linear age. Only the age^2^ results were interpreted in these analyses, and within-group correlations with age^2^ and age^2^-by-diagnosis interaction were examined (Fig. 3C and D). Age^2^-by-diagnosis results were again masked with within-group age^2^ correlations at z > 1.7 (i.e., ASD > TD Age^2^ contrast at z > 2.3 was masked by regions where neural activity showed positive correlations with age^2^ in ASD at z > 1.7 and by regions where activity showed negative correlations with age^2^ in TD at z > 1.7). To further visualize the age-neural activity relationship, parameter estimates were extracted from representative clusters in the ASD > TD Age^2^ contrast masked by within-group age^2^ correlations (i.e., Fig. 3C, bottom row) and plotted against age (Fig. 3D).

IQ and mean absolute motion were regressed out of parameter estimates in the scatter plots (i.e., Fig. 3B and 3D) to accurately represent the neuroimaging analyses.

#### Age*SOR interaction effect on sensory-evoked neural activation in ASD

To test whether SOR severity moderated the effect of age on neural activation in ASD youth, we ran within-group analyses with an interaction term (age*SOR). The FEAT analyses included an intercept term (i.e., column of 1 s), SOR severity, age and the interaction term as well as mean absolute motion as a covariate of no interest. Both positive and negative interactions between age and SOR were examined. To visualize and interpret the direction of the neural activation-age-SOR relationship in significant interaction clusters, parameter estimates were extracted from these clusters (Fig. 4A) and plotted against age by high and low SOR groups (Fig. 4B). The ASD group was divided into high and low SOR groups with a median split, *for visualization purposes only,* in Fig. 4B.

To examine an interaction effect of SOR severity and age^2^ on neural activity, a separate analysis was conducted with additional age^2^ and age^2^*SOR terms where only age^2^*SOR results were interpreted. As described above, both positive and negative interactions between age^2^*SOR were interrogated, and parameter estimates were extracted from activation clusters and plotted against age in low SOR and high SOR groups.

Mean absolute motion was regressed out of parameter estimates in the scatter plots (i.e., Fig. 4B and D) for consistency with the neuroimaging analyses in Figs. 4A and C.

To control for any possible effects of sex, we repeated all of our analyses (i.e., analyses represented in Figs. 2, 3 and 4) with sex as a covariate of no interest. All analyses remained consistent after controlling for sex, except for one interaction analysis which is reported in the Additional file 1: Figure S6.

## Results

### Determination of the final sample

Data were initially collected for 63 ASD and 47 TD participants. Participants who had more than 1 mm mean absolute motion were excluded from analyses (8 ASD and 4 TD). An additional 3 ASD and 2 TD participants were excluded due to being a sibling of another participant (1 ASD), a structural abnormality (1 TD), and equipment failure or artifacts (2 ASD, 1 TD).

### Behavioral results

**Fig. 1:**
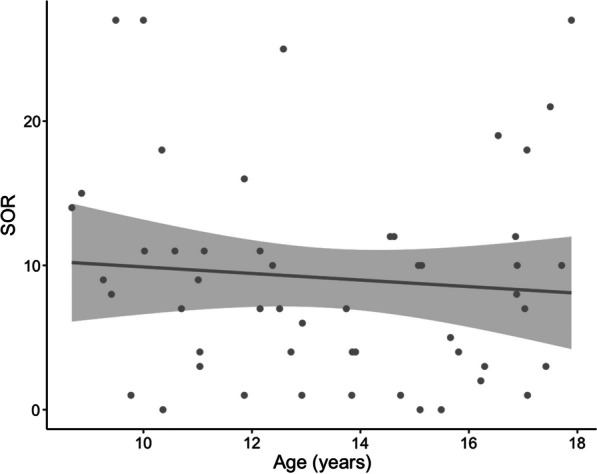
Relationship between age and SOR severity in ASD youth. No significant correlation was found between age and SOR severity in the ASD group (r = − 0.09, *p* = 0.54)

Age was not significantly correlated with SOR severity in our ASD sample (*r* = − 0.09, *p* = 0.54, 95% confidence interval = (− 0.35, 0.19); Fig. 1).

### Neural response to sensory stimulation in older and younger ASD and TD youth

#### Pre-teens

**Fig. 2:**
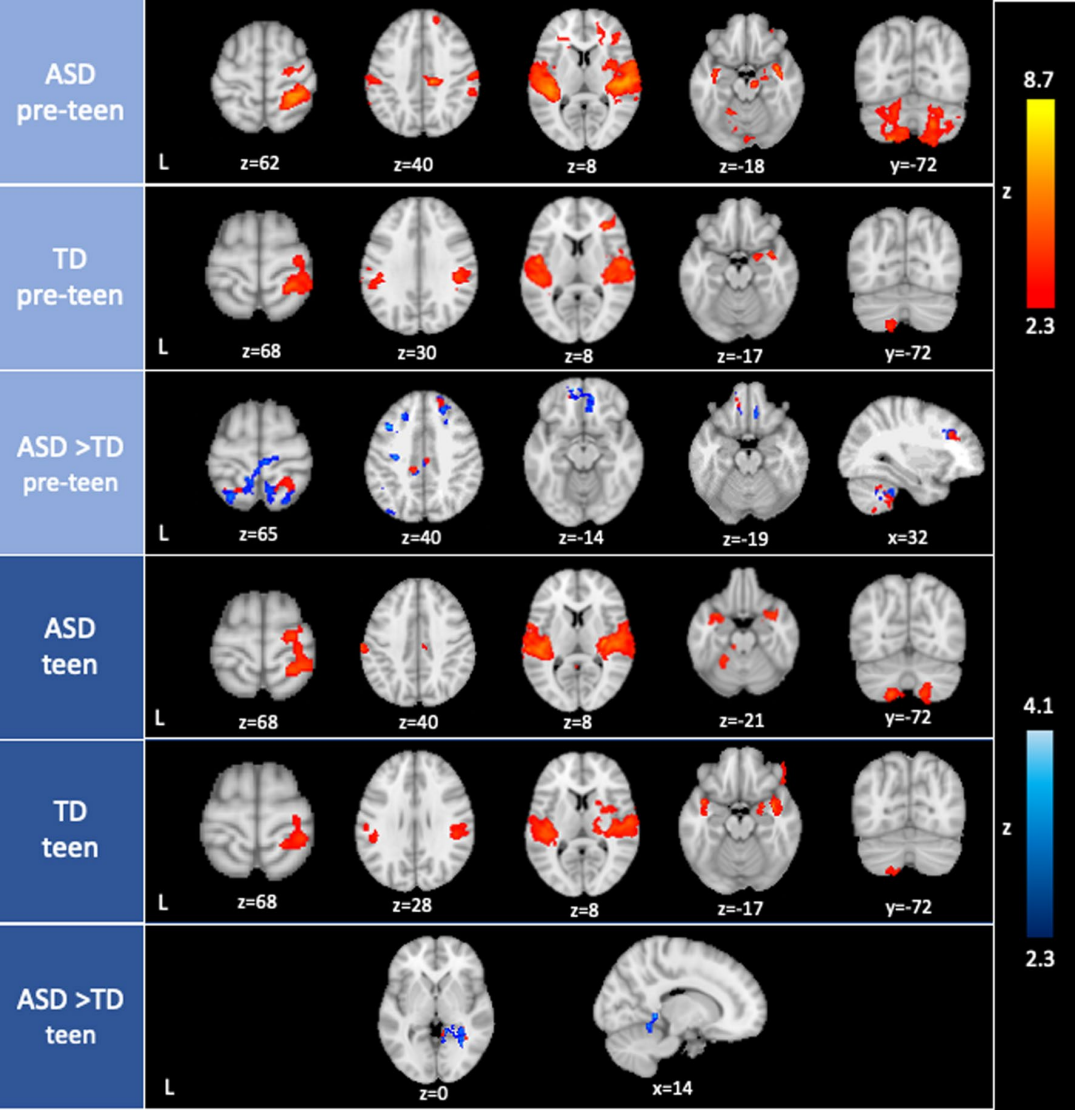
Sensory-evoked neural activation in pre-teens and teens. The top three rows display within- and between-group results in pre-teens (ages 8.6–12.9), and the bottom three rows display results in teens (ages 13.6–18.0). Between-group contrasts were masked with within-group results at z > 1.7, with red indicating clusters that were activated in the ASD group (ASD > TD) and blue showing clusters that were deactivated in the TD group (TD < ASD). There were no TD > ASD results that survived the statistical threshold in pre-teens and teens. *ASD* Autism spectrum disorder, *TD* Typically developing youth

Both ASD and TD pre-teens showed activation in widespread sensory processing and frontal regions in response to the joint sensory stimulation, including auditory temporal cortex, sensorimotor cortex, insular cortex, right amygdala, frontal cortex, temporal pole, superior parietal lobule, anterior supramarginal gyrus, as well as left posterior cerebellum (VIIB, VIIIA and VIIIB) and left cerebellar Crus II (Fig. 2; Table 2).

In addition to these shared neural responses, ASD pre-teens further activated the posterior cingulate and posterior supramarginal gyrus, several subcortical regions (bilateral hippocampus, right thalamus and right putamen), and the brainstem. The cerebellum displayed bilateral activation in ASD pre-teens, additionally including left lobules V and X; bilateral lobule VI and Crus I; and right lobules VIIB, VIIIA, and Crus II. A direct between-group comparison indicated that ASD pre-teens showed significantly greater activity than TD pre-teens in multiple regions including sensorimotor regions (i.e., precentral and postcentral gyri, superior parietal lobule), precuneus, frontal cortex (i.e., ventromedial PFC (vmPFC), dorsolateral PFC (dlPFC) and frontal pole), superior lateral occipital cortex, angular gyrus, anterior and posterior cingulate gyrus, and right cerebellum (lobules I-VI, VIIB and VIIIA, and Crus I and II). Between-group differences in most of these regions stemmed from both heightened neural activation in ASD pre-teens and enhanced deactivation (i.e., reduced response to sensory stimuli compared to fixation) in TD pre-teens (Fig. 2; Table 2). We found no significant TD > ASD differences in pre-teens.

#### Teens

Similar to pre-teens, both ASD and TD teens displayed sensory-evoked activation in widespread sensory processing and subcortical regions including auditory temporal cortex, sensorimotor cortex, insular cortex, amygdala, putamen, temporal pole, superior parietal lobe, anterior supramarginal gyrus, and left posterior cerebellum (VIIB, VIIIA and VIIIB; Fig. 2; Table 2).

ASD teens further activated the superior frontal gyrus, posterior cingulate gyrus, brainstem, posterior parahippocampal gyrus, and bilateral cerebellar Crus II, right lobule VIIB and VIIIA, and left cerebellar lobules V, VI and X. TD teens showed activation in OFC/frontal pole, right thalamus, right pallidum, and cerebellum (left Crus I and II). A direct between-group comparison showed that there were some significant group differences, though much less so than in the pre-teen comparison: ASD compared to TD teens showed significantly greater activity in a cluster covering lingual gyrus, posterior cingulate gyrus, right hippocampus/thalamus and right cerebellar lobule V. These differences were primarily driven by enhanced deactivation in TD teens (Fig. 2; Table 2). There were no significant TD > ASD differences in teens.

### Age correlations with sensory-evoked neural activation

**Fig. 3:**
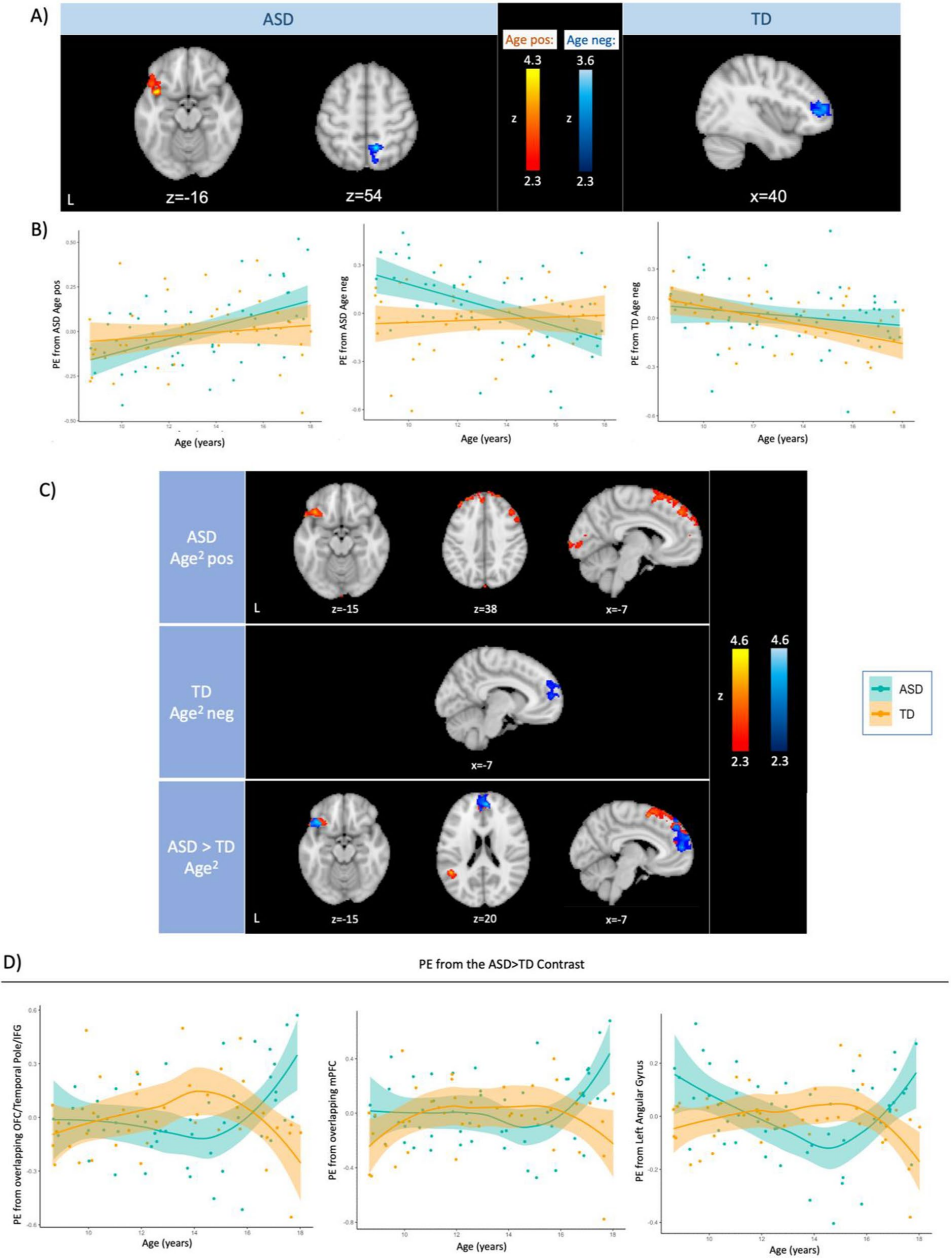
Age correlations with sensory-evoked neural activation.** A** Brain regions where age correlated significantly with neural activation in response to joint (i.e., tactile and auditory) sensory stimulation in ASD youth (left) and TD youth (right). There were no significant positive age correlations with neural activity in TD and significant age-by-diagnosis interaction clusters. **B** Scatter plots of parameter estimates extracted from the significant clusters in A) plotted against age. **C** Brain regions where age^2^ (i.e., quadratic age) correlated significantly with sensory-evoked neural activation. There were no significant clusters where age^2^ correlated with neural activity negatively in ASD and positively in TD. *Top:* positive age^2^ correlations with neural activity in ASD. *Middle*: negative age^2^ correlations with neural activity in TD. *Bottom:* regions where age^2^ showed a more positive correlation with neural activation in ASD than TD. ASD > TD results were masked by ASD Age^2^ positive (red) and TD Age^2^ negative (blue) results at z > 1.7. **D** Parameter estimates were extracted from representative clusters from the ASD > TD Age^2^ contrast (i.e., the bottom row of C)) and plotted against age. Representative clusters were *(left)* the OFC/temporal pole/IFG cluster where red (i.e., ASD > TD Age^2^ masked by ASD Age^2^ pos) overlapped with blue (i.e., ASD > TD Age^2^ masked by TD Age^2^ neg), *(middle)* the mPFC cluster where red and blue overlapped; and *(right)* left angular gyrus cluster. IQ and mean absolute motion were regressed out of parameter estimates in scatter plots in B) and D). *Age pos* regions where age positively correlated with neural activation, *Age neg* regions where age negatively correlated with neural activation, *ASD* Autism spectrum disorder, *TD* Typically developing youth, *PE* Parameter estimates, *OFC* Orbitofrontal cortex, *IFG* Inferior frontal gyrus, *mPFC* Medial prefrontal cortex

We next investigated how neural activation during sensory stimulation differed as a function of age in ASD and TD youth by entering age as a bottom-up regressor in a whole-brain analysis. In ASD children, increased age was correlated with stronger activation in a cluster covering insular cortex/temporal pole/OFC, but weaker activation in a cluster covering precuneus/superior lateral occipital cortex during sensory stimulation (Fig. 3; Table 2). In TD youth, age was negatively correlated with activation in frontal pole/ventrolateral PFC (vlPFC). Parameter estimates from each of these clusters were extracted and plotted to visualize the age*neural activation relationship in both ASD and TD groups. There was no significant age-by-diagnosis interaction effect.

We also examined a nonlinear relationship between age and neural activity by entering age^2^ as a bottom-up regressor (Fig. 3C and D). In ASD, age^2^ was positively correlated with neural activation in the medial prefrontal cortex (mPFC), left and right dlPFC, OFC/temporal pole/inferior frontal gyrus (IFG), and occipital cortex. In TD, age^2^ was negatively correlated with neural activation in the mPFC. There were no significant negative age^2^ correlations in the ASD or positive age^2^ correlations in the TD group.

We found that neural activity in the mPFC, OFC/temporal pole/IFG and left angular gyrus was correlated more positively with age^2^ in ASD compared to TD youth. Some of these ASD-TD differences were driven by an enhanced positive correlation between activation and age^2^ in ASD (e.g., angular gyrus), while some were driven also by a stronger negative association between neural activity and age^2^ in TD youth (e.g., mPFC).

### Age*SOR interaction effect on sensory-evoked neural activation in ASD

**Fig. 4:**
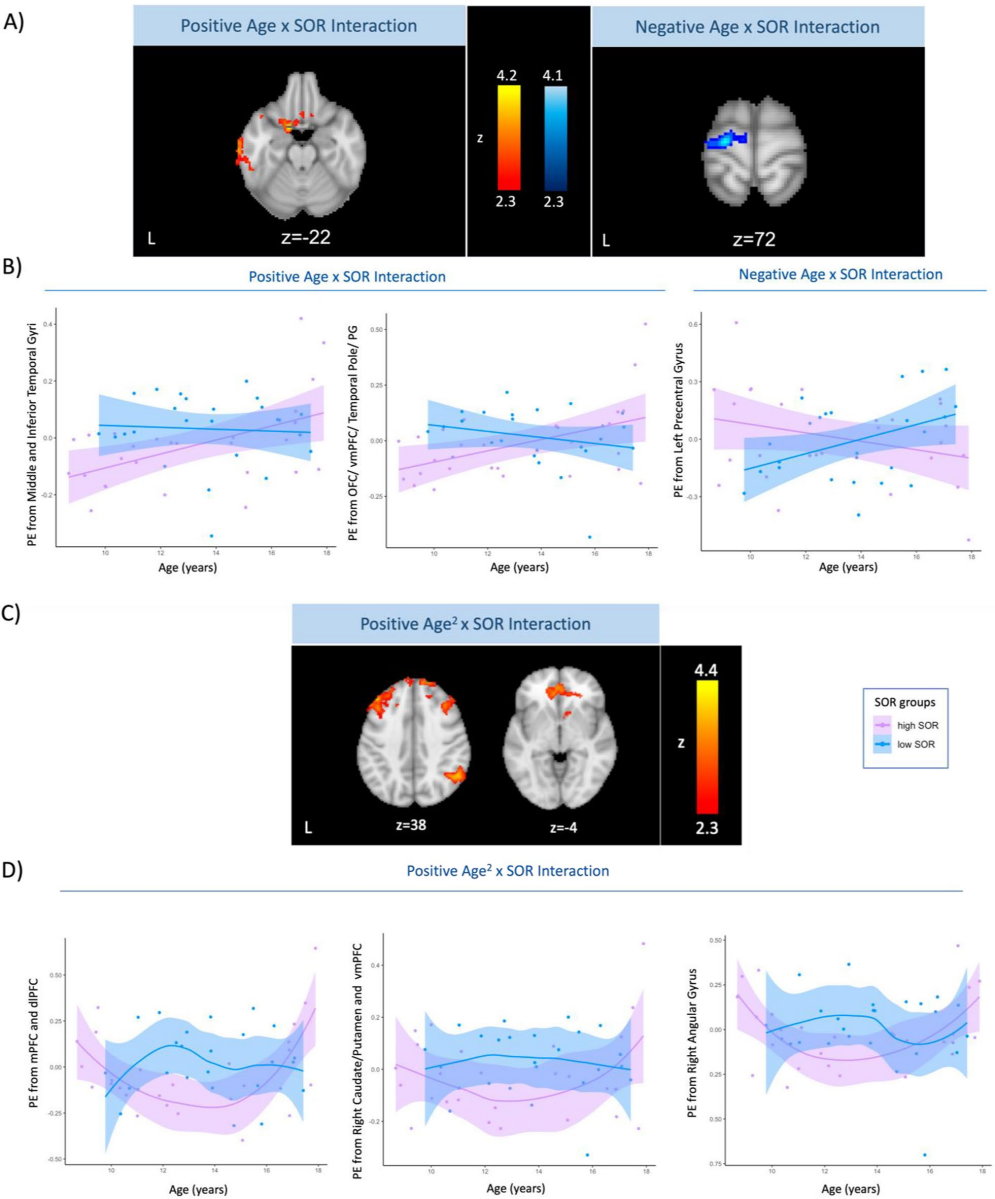
Age*SOR interaction effects on neural activation. **A** Clusters displaying *(left)* positive and *(right)* negative age*SOR linear interaction effects on the neural response to sensory stimulation. **B** Parameter estimates extracted from the significant interaction clusters in A) plotted against age by SOR group. High and low SOR groups (i.e., more and less severe SOR, respectively) were created with a median split within ASD participants for scatter plots solely to visualize the results displayed in A). **C** Regions showing a positive age^2^*SOR interaction effect on sensory-evoked neural activity. There were no significant negative age^2^*SOR interaction clusters. **D** Parameter estimates extracted from the clusters in C) were plotted against age^2^*SOR severity group as described in B) above. Mean absolute motion was regressed out of parameter estimates in the scatter plots in B) and D). *PE* Parameter estimates, *SOR* Sensory over-responsivity, *OFC* Orbitofrontal cortex, *PG* Parahippocampal gyrus, *vmPFC* Ventromedial prefrontal cortex, *mPFC* Medial prefrontal cortex, *dlPFC* Dorsolateral prefrontal cortex

We next investigated whether the effect of both linear and nonlinear age on neural responses to sensory stimulation varied as a function of SOR severity within the ASD group. We examined both a positive and negative interaction between linear age and SOR severity: The positive interaction results indicated brain regions where neural activation was *positively* associated with age for children with more severe SOR. The negative interaction results displayed regions where neural activation was *negatively* associated with age for children with more severe SOR. We found a positive age*SOR interaction effect on neural activation in middle/inferior temporal gyri, as well as in a frontal cluster covering OFC and vmPFC as well as extending to temporal pole and anterior parahippocampal gyrus (Fig. 4A, left), such that ASD youth with more severe SOR showed greater increases in neural activation with age in these regions. Furthermore, there was a significant negative age*SOR interaction effect such that ASD youth with higher SOR displayed greater age-related reductions in left precentral gyrus activity (Fig. 4A, right).

We found that a nonlinear age interaction with SOR (age^2^*SOR) further predicted sensory-evoked neural activation (Fig. 4C and D). There was a positive interaction of age^2^ with SOR relating to neural activation in the mPFC, dlPFC, right caudate/right putamen/vmPFC, and right angular gyrus. There were no significant negative age^2^*SOR interaction clusters. 

## Discussion

The purpose of the current study was to investigate the influence of age on neural responses to sensory stimulation. We also assessed whether SOR severity impacted the effect of age on sensory-evoked neural activity in autism. We found that while ASD youth did show hyperactivation to sensory stimulation as shown in prior studies [24, 25], this hyperactivation compared to TD youth was mainly seen in the younger, pre-teen age group. Age was associated with increased activation in sensory integration and emotion regulation regions in ASD, particularly during late adolescence, suggesting that delayed maturation of sensory regulation networks may underlie SOR in younger children but may also be a potential mechanism for reduced neural hyperactivation with age. Furthermore, these age-related increases in activity in emotion regulation regions were stronger for ASD youth with higher SOR, indicating that SOR may be associated with protracted maturation of sensory regulation networks.

### Neural differences in older and younger ASD and TD youth

ASD pre-teens displayed hyperactivity in neural responses to sensory stimuli across multiple brain regions compared to TD pre-teens in regions consistent with previous findings (e.g., sensorimotor and frontal regions; [18, 19]). Notably, ASD pre-teens also showed relative hyperactivation in several cerebellar areas, and while such hyperactivation has been reported previously [25], it was relatively de-emphasized compared to findings in sensory-limbic regions. The cerebellum is hypothesized to play a critical role in maintaining prediction models and to signal errors when sensory consequences do not meet the predicted outcomes [42]. Thus, more focus on the cerebellar role in atypical sensory processing may be necessary, particularly in pre-teen ASD youth. In contrast with pre-teens, we found few neural differences between ASD and TD teens. These results suggest that previously reported neural hyperactivation in ASD youth may be driven by pre-teens and highlights the importance of accounting for age while investigating neural correlates of sensory features in autism.

### Associations between age and neural responses to sensory stimulation

Importantly, we found that reactivity in some key sensory processing and regulation regions varied continuously as a function of age in ASD, suggesting a mechanism for developmental changes in sensory reactivity. Our results indicate that, as they get older, ASD youth increase engagement in frontal regions, such as the OFC and mPFC, and in regions important to sensory interpretation such as the temporal pole and insular cortex in response to aversive sensory stimulation. In particular, nonlinear analyses demonstrated that activity in these regions increases later in ASD compared to TD (i.e., increased steeply around 14–15 years in ASD with an inverse U-shape-like trend in TD – increasing in late childhood/early teens and then decreasing), suggesting a delayed functional maturation of the frontal cortex in sensory processing in autism. Critically, the OFC has been implicated as an important region in sensory regulation in multiple studies and as a differentiating mechanism between ASD youth with high and low SOR [23, 25]. Our results strengthen these findings and suggest that the OFC may be part of the neural mechanism that underlies developmental changes in sensory reactivity in autistic youth. Similarly, the temporal pole is involved in the emotion regulation network [43], whereas the insular cortex receives input from sensory regions across modalities and is involved in emotional evaluation of sensory signals [44, 45]. Increased engagement of these three regions indicates that, as ASD youth age, they may be better able to recruit neural networks for emotional evaluation and regulation to reassess and downregulate their neural responses to bothersome sensory signals.

We also found that activation in the occipital lobe decreases with age in ASD. Green et al., [23] previously reported atypical increase in visual cortex activation during sustained joint auditory and tactile activation in ASD youth with high SOR. Given that our fMRI task does not include visual stimuli, activation in visual regions suggests a lack of differentiation in sensory signals, and results from the current study indicate that this differentiation may improve with age.

Despite increases in OFC activation with age in ASD, TD youth showed a negative relationship between age and activity in the frontal pole/vlPFC. Prior evidence suggests that once amygdalar downregulation is over-learned, prefrontal engagement may not be required to regulate responses to emotional stimuli in adults [46]. Thus, age-related decreases in frontal cortex activity in TD youth may indicate that sensory regulation becomes over-learned and thus requires less cognitive effort as TD youth age.

### SOR severity impacts the effect of age on sensory-evoked neural responses in ASD

In the current study, we investigated whether SOR severity moderated the effect of age on sensory-evoked neural responses in ASD. We showed that ASD youth with more severe SOR displayed greater age-related increases in neural activity in key sensory regulatory regions, namely the OFC and vmPFC as well as the temporal pole and anterior parahippocampal gyrus. Moreover, when examining the nonlinear effect of age, we found that rate of change in neural activity differed among ASD youth based on SOR severity in a number of frontal regions as well as in the right caudate/putamen and right angular gyrus, with increases in mid-adolescence for those with higher SOR. This suggests that the age-related increases in frontal cortex that we found in our autism group may be driven by those with more severe SOR, which is consistent with the idea that these networks are more delayed and mature later specifically for those individuals with SOR. Somewhat similarly to the OFC, the vmPFC is also implicated in developmental improvements in emotion regulation and functionally matures in its role of downregulating the amygdala during early adolescence [27, 29]. Thus, for some ASD youth with elevated SOR, regulatory mechanisms through the OFC and vmPFC may be increasingly recruited with age, potentially providing a compensatory mechanism to allow individuals to carry out daily-life functions despite atypical sensory processing. This could represent a delayed or more prolonged period of sensory regulatory development for autistic youth with more severe SOR, compared to TD youth or ASD youth without SOR who may develop these regulatory mechanisms earlier in childhood. To note, such improvements in neural regulation may not necessarily mean that the discomfort from sensory signals fades away with age, particularly given that, behaviorally, SOR did not decline with age in our sample despite the neural findings. Instead, our results suggest that ASD youth may get better at coping with aversive sensory stimuli through enhanced engagement of compensatory regulatory mechanisms with age. Furthermore, the absence of a significant relationship between age and parent-reported SOR in our study may also be due to interindividual heterogeneity in developmental trajectories of sensory features in autism [17], with a subset of our participants improving in SOR, while others remain stable or worsen with age. Future longitudinal research will be instrumental in elucidating whether the reported associations between age, SOR and neural responsivity are specific to ASD youth showing developmental improvements in SOR.

We also found that ASD youth with higher SOR additionally show greater age-related increases in the middle and inferior temporal gyri, which relate to sensory association functions such as multisensory integration and tactile object recognition [47–49]. Increased activation in these regions with age in ASD youth with more severe SOR may signify a delayed ability to characterize and contextualize sensory information, which improves with age.

We further identified a negative interaction between SOR and age in the *left* sensorimotor cortex in ASD, indicating that youth with higher SOR decrease reactivity in this region more as they age. Tactile stimulation was administered on the left arm, which would typically be processed in the *right* sensorimotor cortex. Contralateral sensorimotor cortex activity has previously been associated with high SOR in ASD [23], and taken together with the results showing engagement of the occipital cortex during auditory and tactile stimulation, these findings support the idea that neural mechanisms for sensory processing may be less segregated in ASD. Our data indicate that the engagement of these unrelated sensory regions may be heightened earlier in childhood in ASD youth with more severe SOR and decrease with age across the teen years, potentially also contributing to improvements either in SOR or in coping with aversive stimuli.

In summary, in the current study, we showed that neural hyperactivity in response to sensory signals in ASD youth improves with age in ASD. Our results support prior findings implicating the frontal cortex, and especially the OFC, as a key sensory regulatory region, and further indicate that the OFC may at least partially underlie age-related reductions in neural sensory hyperreactivity. Our investigation has multiple strengths, including studying sensory responsivity with mildly aversive (yet tolerable) stimuli to mimic real-life SOR experience, taking both a categorical and continuous approach to investigate the effects of age on neural responses to sensory stimuli in ASD, examining age-related changes both linearly and quadratically, and involving a fairly diverse sample that is representative of the population in the metropolitan setting where the study was conducted. Nevertheless, our study has several limitations that should be considered in interpreting our findings.

### Limitations and future directions

The current investigation is a cross-sectional study and does not track developmental changes in sensory features within-individuals. Recent longitudinal investigations show evidence for heterogeneity in developmental trajectories of sensory features in autism [17]. Future longitudinal studies should examine changes in behavioral SOR developmentally as well as study neural responses to sensory information longitudinally to characterize differences in age-related changes in neural responses based on developmental trajectories (i.e., showing developmental improvement, worsening, or stability of SOR symptoms). Because the current study utilized task-based fMRI, we were not able to include ASD youth below borderline intellectual functioning or with minimal verbal skills. Notably, Jung et al., [34] linked sensory-evoked neural activity to heart rate and skin conductance responses, suggesting that physiological measures could help generalize this research to a wider range of the autism spectrum. In our categorical analyses, we defined developmental groups based on chronological age as pre-teens and teens. Dividing a pediatric sample by age is a crude measure that does not consider puberty and overall hormonal changes that accompany this developmental stage. Although, to our knowledge, there is no current evidence on puberty affecting sensory responsiveness, future research should examine how pubertal status relates to changes in neural circuits associated with sensory responsivity. Similarly, sex differences in developmental trajectories of SOR should be interrogated in future studies, with a larger sample. While previous research found no differences in parent-reported SOR severity in ASD females and males, neural mechanisms of SOR showed differences based on sex [33], which may underlie development of distinct compensatory mechanisms in females and males as ASD youth age. Moreover, in the current study, we use parent-reported SOR as our behavioral SOR measure. While using parent-reported sensory responsivity data has many advantages, such as reflecting the child’s responses in a general and context-sensitive manner [50], recent research showed that observed (but not parent-reported) SOR improved with age in ASD [35]. These findings indicate that observation-based assessments may better capture developmental improvements in children’s ability to regulate their sensory reactivity, while parent-report may reflect how ASD youth *feel* about the sensations. This may be another explanation for the lack of a significant association between age and behavioral SOR in the current investigation, as parents may be capturing their child’s continued dislike of sensory stimulation rather than any recent improvements in SOR-related behavior. Thus, future studies should consider using observed SOR assessments in addition to parent-reports to track behavioral changes in SOR. To note, in the current study, the sensory stimulation introduced to the participants were of non-social nature. Autistic youth may process non-social sensory information distinctively compared to their TD peers [51, 52]. Whether and how responsivity to social and non-social sensory information differs in autistic youth remain an empirical question and should be addressed in future investigations. Lastly, to consolidate our findings implicating a role for the OFC in age-related changes in sensory reactivity, future research should investigate a causal link between these frontal mechanisms and sensory regulation in ASD (e.g., through animal model research or through analytic methods such as Granger causality or dynamic causal modeling), and explore these mechanisms as a potential therapeutic target for SOR.

## Conclusion

In the current study, we investigated how age relates to neural responses to sensory stimulation in ASD youth and examined whether SOR severity influences the effect of age on neural responsivity. Compared to TD youth, younger ASD youth showed widespread neural hyperactivation to sensory stimuli, particularly in sensorimotor, frontal and cerebellar regions. However, there were few diagnostic group differences in neural responses in older, teen-aged youth. In ASD, older age was associated with increased recruitment of emotion regulation and sensory integration regions and decreased involvement of task-unrelated sensory regions. Neural activity in emotion regulation regions such as the frontal cortex showed a distinct age-related trajectory in ASD youth. The effect of age on neural sensory responses was moderated by SOR severity, such that these age-related changes were stronger for those with higher SOR. These results indicate that, although the maturation of regulatory mechanisms might be protracted in ASD, compensatory frontal mechanisms may develop with age to support sensory regulation in autistic youth. From a research perspective, our investigation highlights that age should be taken into consideration in studying the neural correlates of sensory features in autism, as younger ASD youth may be driving the findings in prior SOR studies. From a therapeutic perspective, our findings indicate that behavioral interventions that aim to strengthen cognitive regulation mechanisms may be an effective treatment avenue for SOR in autism.

### Supplementary Information

**Additional file 1.** Supplementary Tables and Figures.

## Data Availability

The datasets used and/or analyzed during the current study are available from the corresponding author on reasonable request.

## Abbreviations

ASD: Autism spectrum disorder
TD: Typically developing youth
SOR: Sensory over-responsivity
fMRI: Functional magnetic resonance imaging
PFC: Prefrontal cortex
OFC: Orbitofrontal cortex
F: Female
ADI-R: Autism Diagnostic Interview-Revised
ADOS-2: Autism Diagnostic Observation Schedule - second edition
FSIQ: Full-scale IQ
WASI: Wechsler Abbreviated Scales of Intelligence
SP3D: Sensory Processing 3-Dimension Scale Inventory
FSL: FMRIB Software Library
FEAT: FMRI Expert Analysis Tool
FLAME: FSL’s Local Analysis of Mixed-Effects State
FWE: Family-wise error
Pre-teens: Pre-teenage children
Teens: Teenagers
vmPFC: Ventromedial prefrontal cortex
dlPFC: Dorsolateral prefrontal cortex
vlPFC: Ventrolateral prefrontal cortex
IFG: Inferior frontal gyrus
