# Supplementary material for: Age-related changes in neural responses to sensory stimulation in autism: a cross-sectional study
Source: Mol Autism. 2023 Oct 11;14:38. doi: 10.1186/s13229-023-00571-4 (PMC10566124; doi:10.1186/s13229-023-00571-4)
Supplement: Supplementary file 1 — Additional file 1. Supplementary Tables and Figures. [file 13229_2023_571_MOESM1_ESM.docx]

**SUPPLEMENTAL INFORMATION**

- 1. Medications and comorbidities.

**Supplemental Table 1. Psychotropic Medications**

|  | ASD | TD |
| --- | --- | --- |
| No medications | 29 | 39 |
| SSRI | 10 | 0 |
| Stimulant | 15 | 0 |
| α2 Agonist | 7 | 0 |
| Antipsychotic | 5 | 0 |
| Anticholinergic | 1 | 0 |
| Unknown | 1 | 2 |

ASD: Autism Spectrum Disorder; TD: typically-developing youth, SSRI: Selective serotonin reuptake inhibitor.

In total, 22 out of 52 ASD participants were on medication. Some participants were on multiple medications, and thus, the total number in the ASD column above adds up to more than 52. Notably, none of the main study findings (i.e., brain regions where age related to neural activation) significantly differed between ASD participants on and off medication.

**Supplemental Table 2. Co-occuring psychiatric conditions in the ASD sample**

|  | Number of participants |
| --- | --- |
| No co-occurring diagnoses^1^ | 27 |
| Attention-deficit/hyperactivity disorder (ADHD) | 15 |
| Depression | 4 |
| Anxiety | 7 |
| Obsessive-Compulsive Disorder (OCD) | 1 |
| Disruptive Mood Dysregulation Disorder | 1 |
| Speech or reading disorder | 1 |
| Sleep disorder | 1 |
| Unknown | 3 |

^1^This table includes only diagnoses, however co-occuring conditions in autism are commonly underdiagnosed, or may present subclinically, thus this table maybe under-representative of true co-occurring conditions.

- 1. Analyses thresholded at z>2.7

All the brain analyses were repeated with the same variables and covariates at a more stringent threshold of z>2.7. Between-group contrasts were again masked posthoc with within-group contrasts and within-group correlations with age at z>1.7. Results at z>2.7 were overlapped with results at z>2.3 to provide a comparison.


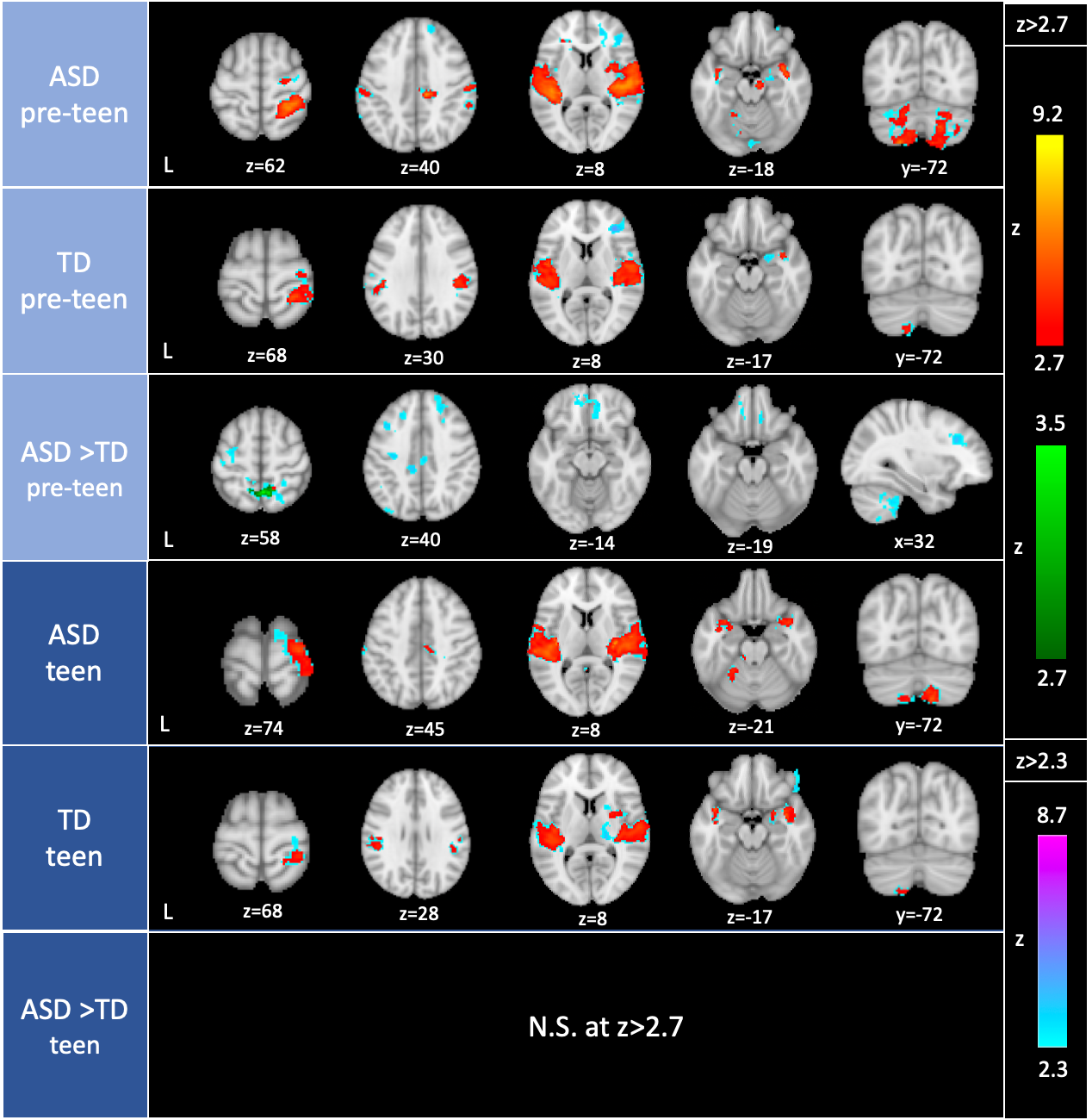


**Supplemental Figure 1.** Sensory-evoked neural activation in pre-teens and teens, thresholded at z>2.7 compared to z>2.3. The top three rows display within- and between-group results in pre-teens, and the bottom three rows display results in teens. Between-group contrasts were masked with within-group results at z>1.7, with red indicating clusters that were activated more in the ASD group and green showing clusters that were deactivated more in the TD group. There were no TD>ASD results that survived the statistical thresholds in pre-teens, and no between-group results that survived the z>2.7 statistical threshold in teens. **red:** *within-group results*: sensory-evoked neural activation, *ASD>TD results:* regions where activation is greater in ASD compared to TD (i.e., ASD>TD pre-teen contrast masked by ASD pre-teen positive contrast); **green:** regions where deactivation is greater in TD compared to ASD youth in between-group results; **blue-purple:** results at z>2.3 (also represented in Figure 2). ASD: Autism Spectrum Disorder; TD: typically-developing youth; N.S.: not significant.

1.
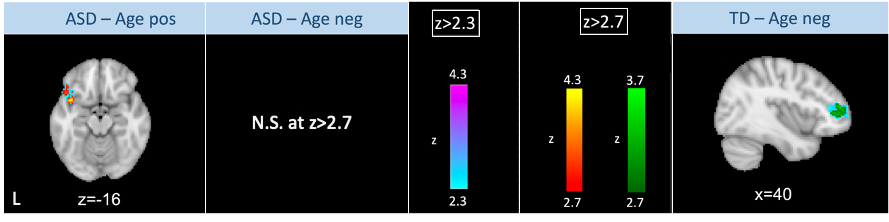


L

1.
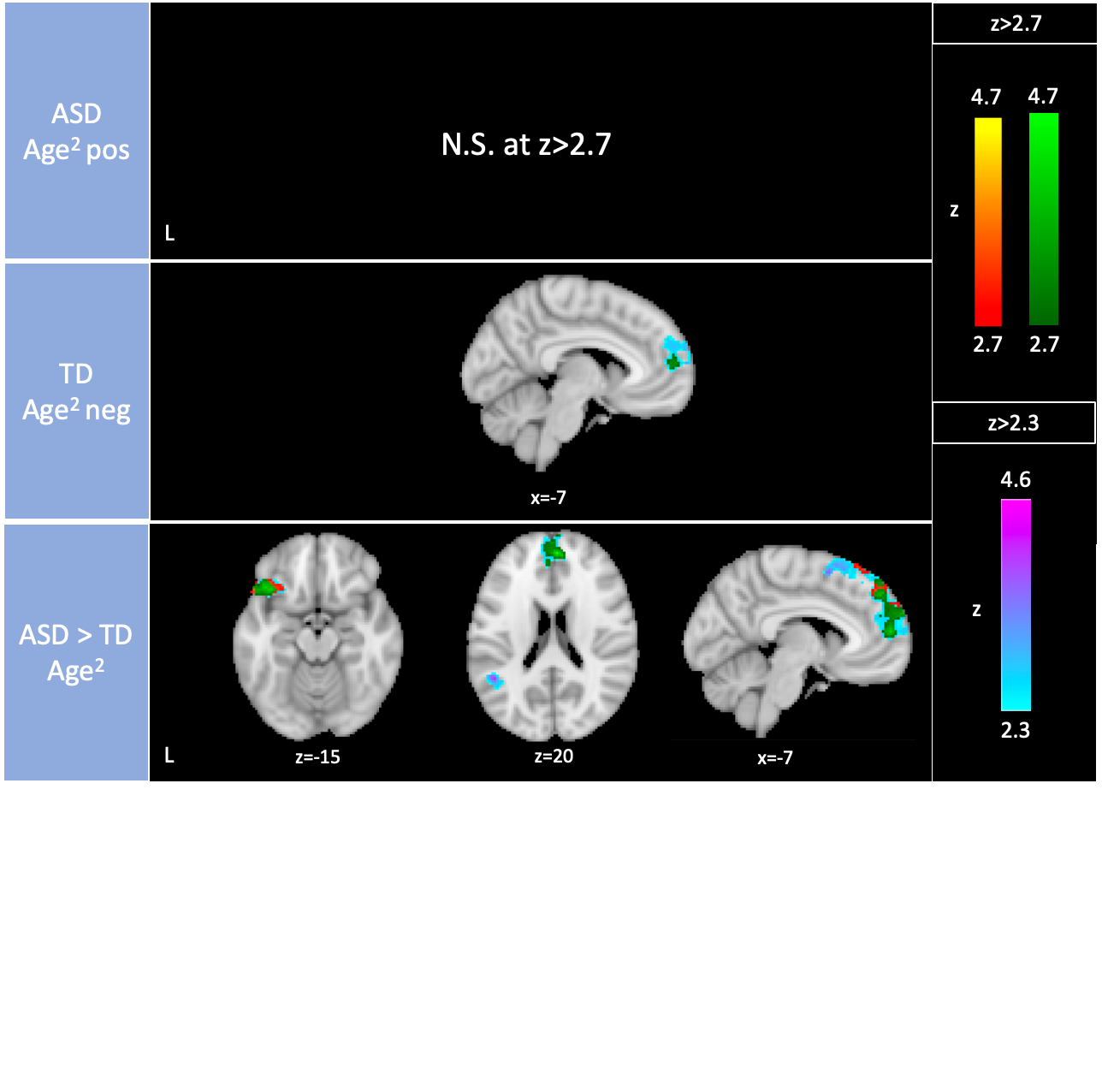


**Supplemental Figure 2.** Age correlations with sensory-evoked neural activation at z>2.7 compared with z>2.3. **A)** Brain regions where age correlated significantly with neural activation in response to joint (i.e., tactile and auditory) sensory stimulation in ASD youth (left) and TD youth (right). There were no significant clusters that correlated negatively with age in ASD at z>2.7. **B)** Brain regions where age^2^ (i.e., quadratic age) correlated significantly with sensory-evoked neural activation. *Top:* there were no positive age^2^ correlations with neural activity in ASD at z>2.7. *Middle*: negative age^2^ correlations with neural activity in TD. *Bottom:* regions where age^2^ showed a more positive correlation with neural activation in ASD than TD. ASD>TD Age^2^ results were masked by ASD Age^2^ pos and TD Age^2^ neg results at z>1.7. **red:** *within-group results:* where age or age^2^ correlated positively with neural activity at z>2.7; *ASD>TD results:* regions where activation is greater in ASD compared to TD (i.e., ASD>TD age^2^ masked by ASD Age^2^ pos) at z>2.7. **green:** *within-group results:* where age or age^2^ correlated negatively with neural activity at z>2.7; *ASD>TD results:* regions where deactivation is greater in TD compared to ASD youth (i.e., ASD>TD age^2^ masked by TD Age^2^ neg) at z>2.7. **blue-purple:** results thresholded at z>2.3 (also represented in Figure 3). Age pos or Age^2^ pos: regions where age or age^2^ correlated positively with neural activity; Age neg or Age^2^ neg: regions where age or age^2^ correlated negatively with neural activity. ASD: autism spectrum disorder; TD: typically-developing youth; N.S.: not significant.


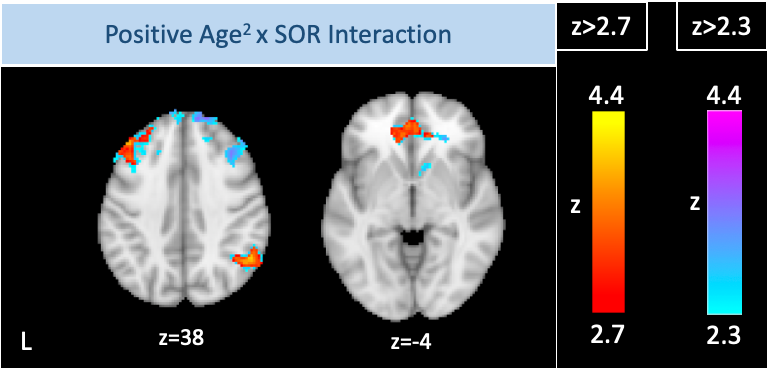


**Supplemental Figure 3:** Age^2^*SOR interaction effect on neural activation in the ASD group, thresholded at z>2.7 compared to z>2.3. Clusters displaying a positive age^2^*SOR interaction effect on the neural response to sensory stimulation. There were no significant age*SOR (i.e., linear) interaction effect clusters. **red:** where age^2^*SOR correlated positively with neural activity at z>2.7; **blue-purple:** results thresholded at z>2.3 (also represented in Figure 4). SOR: sensory over-responsivity.

- 1. Analyses without IQ as a covariate

We previously controlled for IQ in our categorical age analyses (Figure 2) and in analyses where we compared age correlations in ASD and TD groups (e.g., Figure 3C, bottom) because there were significant between-group differences in IQ (Table 1). We repeated all of these analyses without IQ.

The majority of our results without IQ (Supplemental Figure 4 and 5) were consistent with our analyses with IQ, except for the ASD>TD pre-teen contrast. Notably, ASD and TD youth in this age group (i.e., pre-teens) showed significant differences in IQ in our sample (ASD pre-teens X̄ = 106.12; TD pre-teens X̄ = 117.91; *p*=0.008).

We found no significant age*diagnosis interactions in our analyses of linear age correlations with sensory-evoked neural activation, with or without IQ as a covariate.


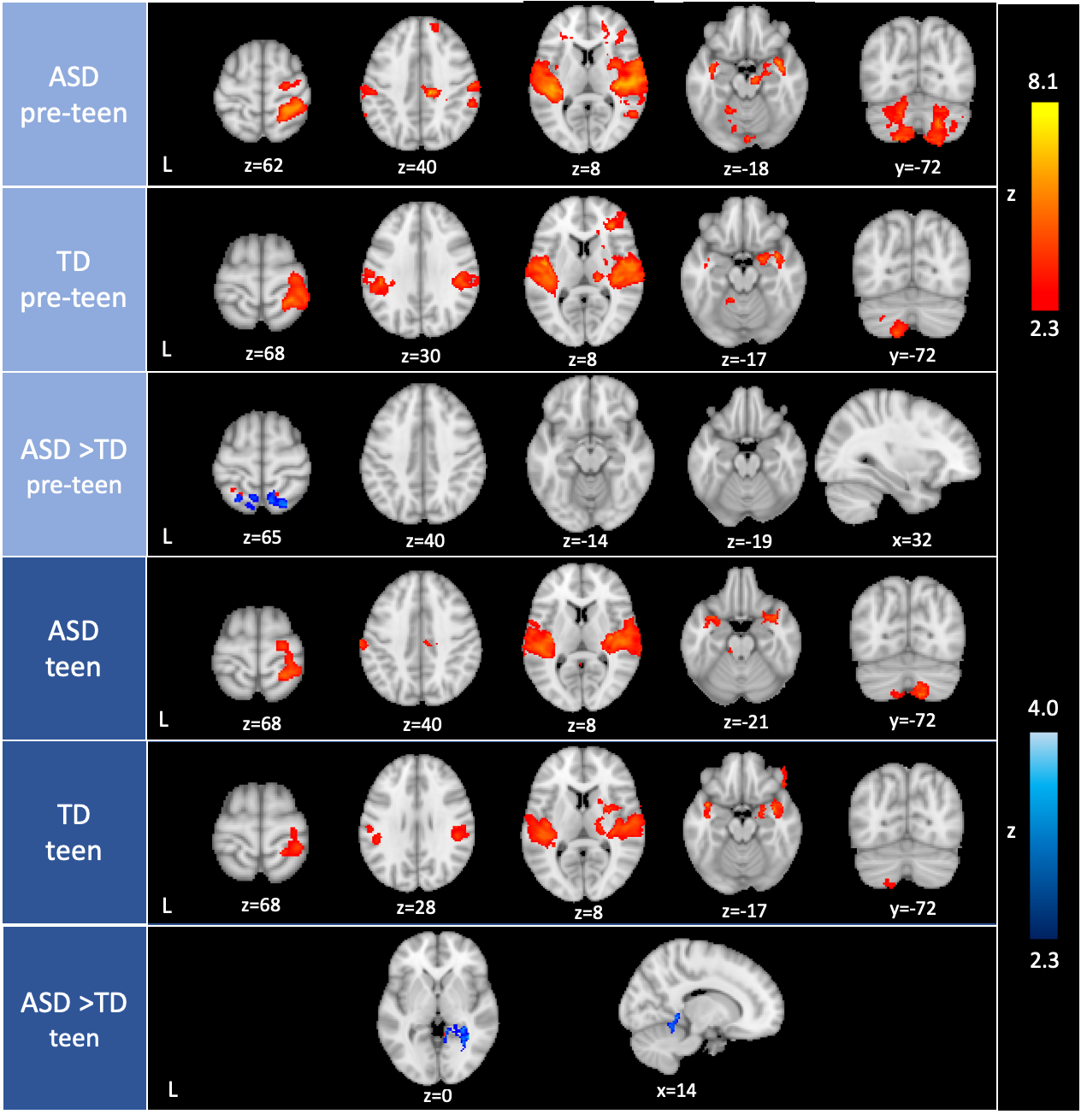


**Supplemental Figure 4.** Sensory-evoked neural activation in pre-teens and teens, without IQ as a covariate. The top three rows display within- and between-group results in pre-teens, and the bottom three rows display results in teens. Between-group contrasts were masked with within-group results at z>1.7, with red indicating clusters that were activated more in the ASD group and blue showing clusters that were deactivated more in the TD group. The same analysis with IQ as a covariate is represented in Figure 2. ASD: Autism Spectrum Disorder; TD: typically-developing youth; N.S.: not significant.


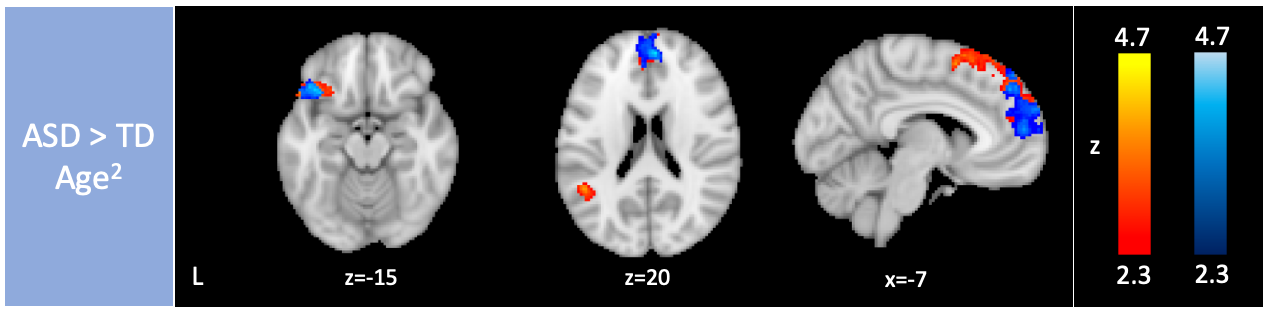


**Supplemental Figure 5.**  Age^2^*diagnosis interaction effect on sensory-evoked neural activation, without IQ as a covariate. The contrast represents regions where age^2^ showed a more positive correlation with neural activation in ASD than in TD. ASD>TD results were masked by ASD Age^2^ positive (red) and TD Age^2^ negative (blue) results at z>1.7. The same analysis with IQ as a covariate is represented in Figure 3C, bottom row. ASD: autism spectrum disorder; TD: typically-developing youth.

- 1. Analyses with sex as a covariate

Analyses were conducted with sex as a covariate to determine if sex might have an effect on any results. Covarying sex had a negligible effect, except for in one analysis: the positive age*SOR interaction analysis results were no longer significant at z>2.3. However, at z>1.7, the clusters emerged again as significant (Supplemental Figure 6), suggesting that including sex may have reduced the power to detect a significant interaction. Notably, parameter estimates extracted from regions of significance did not show significant sex differences (Cluster 1- Middle and Temporal Gyri: ASD Males X̄= -0.014, ASD Females X̄= 0.031; *p*=0.22; Cluster 2- OFC/vmPFC/ Temporal Pole/PG: ASD Males X̄= -0.029, ASD Females X̄= 0.030, *p*= 0.17).


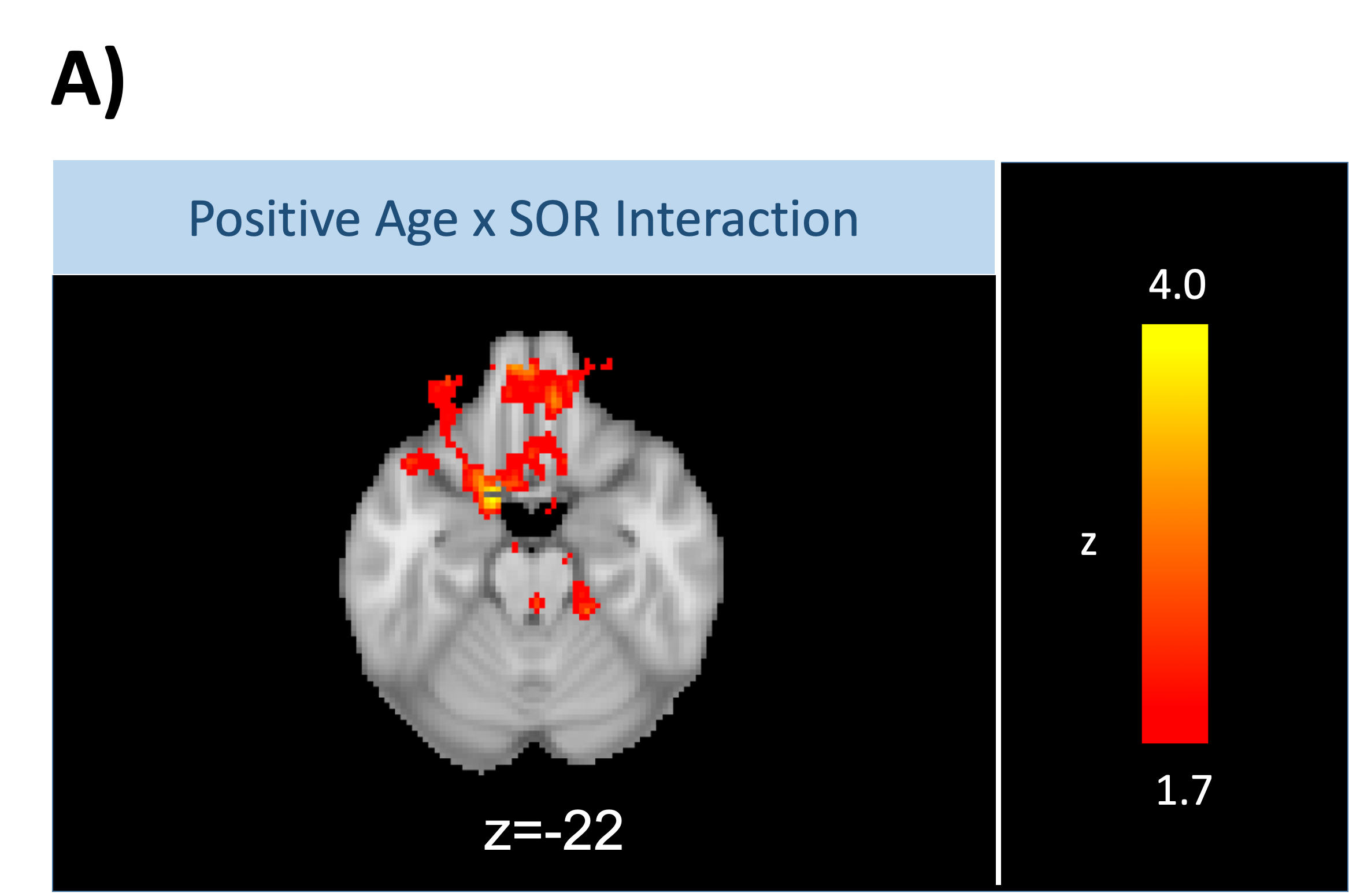


**Supplemental Figure 6.** Positive age*SOR interaction effect on neural activation in the ASD group, controlling for sex. Clusters (red) displaying a positive age*SOR interaction effect on the neural response to sensory stimulation were significant at the threshold of z>1.7. SOR: sensory over-responsivity.
